# Atypical Pattern of Frontal EEG Asymmetry for Direct Gaze in Young Children with Autism Spectrum Disorder

**DOI:** 10.1007/s10803-019-04062-5

**Published:** 2019-05-23

**Authors:** Jenni Lauttia, Terhi M. Helminen, Jukka M. Leppänen, Santeri Yrttiaho, Kai Eriksson, Jari K. Hietanen, Anneli Kylliäinen

**Affiliations:** 1 Faculty of Social Sciences/Psychology, Tampere University, 33014 Tampere, Finland; 2 Faculty of Medicine and Health Technology, Tampere University, 33014 Tampere, Finland; 3 Tampere Center for Child Health Research, Faculty of Medicine and Health Technology, Tampere University, 33014 Tampere, Finland; 4 Department of Pediatric Neurology, Tampere University Hospital, 33014 Tampere, Finland

**Keywords:** Autism spectrum disorder, Intellectual disability, Eye contact, Frontal asymmetry, EEG

## Abstract

This study examined approach-motivation related brain activity (frontal electroencephalogram [EEG] asymmetry) in response to direct and averted gaze in 3- to 6-year-old typically developing (TD) children, children with autism spectrum disorder (ASD), and those with intellectual disability (ID). We found that, in TD children, direct gaze elicited greater approach-related frontal EEG activity than did downcast gaze. This pattern of activity was in contrast to that observed in children with ASD, who showed greater approach-related activity in response to downcast gaze than to direct gaze. ID children did not differ in their responses to different gaze conditions. These findings indicate that another person’s direct gaze does not elicit approach-motivation related brain activity in young children with ASD.

## Introduction

Impairments in social communication skills are one of the earliest signs of autism spectrum disorder (ASD). One striking feature of young children with ASD is that they do not look at other people’s faces and eyes as much as typically developing (TD) children (see e.g., Senju and Johnson 2009). Typically, newborn infants tend to look longer at faces with direct gaze compared to averted gaze (Farroni et al. 2002, 2006) or closed eyes (Batki et al. 2000). Instead, atypical orientation towards faces and eyes can be observed around 1 year of age in children who are diagnosed with ASD in the future (Maestro et al. 2005; Osterling and Dawson 1994; Osterling et al. 2002; Werner et al. 2005). Furthermore, studies on high-risk infants (i.e., infants with an older sibling diagnosed with ASD) have shown that in those diagnosed with ASD in the future, orientation towards the eyes declines gradually during the first year of life (Jones and Klin 2013), and the neural responses to eye gaze are abnormal even before the behavioural symptoms emerge (Elsabbagh et al. 2012).

Despite growing evidence indicating that abnormal processing of direct gaze is an early marker of ASD, the precise nature of the eye-contact difficulties remains unclear. It has been proposed that individuals with ASD are either inattentive to other people’s direct gaze and passively omit eye contact (cf., Dawson et al. 2005), or that they actively avoid looking at others’ direct gaze, that is, avoid making eye contact, due to its aversive nature (Senju and Johnson 2009). The latter hypothesis has been supported by the findings of greater autonomic arousal responses (skin conductance responses, SCRs) to direct gaze in comparison to averted gaze (Kylliäinen and Hietanen 2006; Stagg et al. 2013) or closed eyes (Kylliäinen et al. 2012; Stagg et al. 2013) in 7- to 15-year-old children and adolescents with ASD. In these studies, the researchers suggested that in ASD, the enhanced arousal results in discomfort, and therefore, eye contact is actively avoided.

Enhanced arousal in response to direct gaze has not, however, been a consistent finding in all studies among school-aged children with ASD (cf. Joseph et al. 2008; Kaartinen et al. 2012). Furthermore, Nuske et al. (2015) measured pupil dilation as an indication of arousal response in 2- to 5-year-old children with ASD and age-matched TD children. They found that both groups of children responded with greater pupil dilation in response to direct gaze than in response to averted gaze, indicating no ASD-specific enhanced arousal in response to eye contact. Thus, the heightened arousal in response to direct gaze in 7- to 15-year-old children with ASD (Kylliäinen and Hietanen 2006; Kylliäinen et al. 2012; Stagg et al. 2013) may not be a fundamental reason for the difficulties in making eye contact. Instead, this may be a consequence of diminished exposure to eye contact during development. As these school-aged children were not accustomed to making eye contact in their every-day life, another person’s direct gaze elicited enhanced arousal responses when they were specifically instructed to look at the eyes during the laboratory experiments. Senju (2013) also discussed the possibility that people with ASD do not spontaneously pay adequate attention to social stimuli, such as faces and eyes, but when they are specifically asked to direct their attention to these stimuli, they do not differ from TD people in the way that they perceive social stimuli.

The hypothesis of passive omission of direct gaze in ASD has acquired recent support through studies in young children with ASD. Moriuchi et al. (2017) used eye-tracking as a measure to investigate how 2-year-old TD children, children with intellectual disability (ID), and children with ASD respond to explicit and implicit cueing to look at the eyes. Children with ASD did not look away from the eyes any faster than did TD children when they were explicitly cued to look at the eyes (Moriuchi et al. 2017). The authors concluded that their results supported the presence of passive insensitivity to social signals of others’ eyes, instead of the active avoidance hypothesis. Helminen et al. (2017) reported that young, 2- to 5-year-old children with severe ASD and ID did not show greater attentional orienting responses (measured as heart rate deceleration responses) to direct versus averted gaze, unlike TD children and children with ID but without ASD, which also seems to support the passive omission hypothesis in young children with ASD.

The above results have provided an indirect and inconsistent account of the motivational direction, that is, to approach or to avoid, for direct eye gaze in children with ASD. In order to test the hypotheses regarding passive omission and active avoidance of eye contact in children with ASD, a more direct assessment of the motivational inclinations for eye gaze is needed. A traditional psychophysiological method of studying motivational tendencies is to measure relative frontal asymmetry in electroencephalogram (EEG) alpha-band activity (Davidson 1984; Harmon-Jones and Gable 2018). Studies have shown that, in typical adults, seeing another person’s direct gaze elicits approach-related, relatively greater left-sided frontal activity (left-sided asymmetry), whereas seeing averted gaze elicits avoidance-related, relatively greater right-sided frontal activity (right-sided asymmetry) (Hietanen et al. 2008; Pönkänen et al. 2011; Uusberg et al. 2015). In fact, a previous study investigated frontal EEG asymmetry responses to direct gaze versus closed eyes in school-aged children with ASD (Kylliäinen et al. 2012). This study found greater approach-related, left-sided frontal asymmetry in response to direct gaze versus closed eyes in TD children, whereas there was no difference in frontal EEG asymmetry in response to direct gaze and closed eyes in children with ASD. These findings suggested that, in children with ASD, another person’s direct gaze does not trigger approach motivation. More importantly, their study did not support the hypothesis regarding avoidance-related motivation in response to direct gaze in children with ASD.

In general, previous studies on eye contact in ASD have focused on school-aged, cognitively able children with ASD (as in Kylliäinen et al. 2012), and studies on young children who also have ID are lacking (Itier and Batty 2009; Jack and Pelphrey 2017). However, it is also essential to study children with ASD who have limited verbal communication skills and ID. Research on this group will allow the investigation of the core underlying causes of abnormalities, without the influence of confounding factors such as compensatory mechanisms associated with an advanced intellectual capacity (cf., Jack and Pelphrey 2017) on the findings. The recent findings by Helminen et al. (2017), as described above, gave indirect support for the passive omission of eye contact hypothesis even in young children with ASD and ID. However, without directly measuring the underlying motivational tendencies, it cannot be ascertained whether these children do not exhibit gaze direction-dependent effects in motivation-related brain responses, or whether this group of young children, who often seem to avoid faces/eyes according to the observations of the close ones, would show smaller approach-related responses to direct than to averted gaze.

In the present study, we examined frontal EEG asymmetry responses to direct and averted gaze in 3- to 6-year-old children with severe ASD and ID. TD children and children with ID without ASD served as control groups. Using children with ID as an additional control group, we aimed to control for the effects of a general developmental delay on our findings. In our study, we measured frontal EEG activity whilst the participants were shown videos of faces with direct or downcast gaze or cars, both first static and then moving towards them. The use of motion in the stimuli has helped the children to notice and pay attention to the stimuli in our previous studies (Helminen et al., 2017; Kylliäinen and Hietanen, 2006; Kylliäinen et al. 2012). The dynamicity of social stimuli is also recommended in the ASD-literature (cf., Saitovitch et al. 2013).

Based on earlier results obtained from studies among TD children and adults (Hietanen et al. 2008; Kylliäinen et al. 2012), we hypothesized that TD children and children with ID without ASD would exhibit relatively greater left-sided frontal activity (associated with a tendency to approach) in response to direct gaze than in response to averted gaze. Based on previous studies among school-aged, cognitively able children with ASD (Kylliäinen et al. 2012), one would expect to find no differences between frontal EEG asymmetry responses to direct and averted gaze. The main aim of the present study was to investigate whether a similar pattern would also be observed in children with severe ASD and ID, or whether these children would instead show smaller approach-related activity in response to direct gaze (indicated by less relative left-sided frontal EEG activity in response to direct than to downcast gaze in the children with ASD).

## Methods

### Participants

The study was a part of the Autism and Gaze research project wherein the development of eye contact behaviour was investigated in young children with ASD. Twenty children with ASD, 17 children with ID without ASD and 19 TD children (all between 3 and 6 years in age) who had no history of neurodevelopmental or neurological disorders participated in the study. Children with ASD and children with ID were recruited from the Department of Pediatric Neurology, University Hospital of Tampere, Finland. Children with ID were also recruited from the Outpatient Intellectual Disabilities Clinic, University Hospital of Tampere, and TD children were recruited from local day-care centres. The study was reviewed by the Ethical Committee of the Pirkanmaa Hospital District (ETL R12098). Written informed consent was obtained from the participants’ parents after they received both written and oral information about the study.

The developmental age of ASD and ID children was estimated by a clinical neuropsychologist using the Wechsler Preschool and Primary Scale of Intelligence, third edition (WPPSI-III; Wechsler 2002) and/or Bayley Scales of Infant and Toddler Development II (Bayley 2006). The ASD and ID groups were matched by developmental age, chronological age, and sex. TD children and children with ASD were matched by chronological age and sex. Eight children with ASD, 7 children with ID, and 7 TD children were excluded from the final analysis owing to a lack of cooperation during the experiment, leading to insufficient acceptable data (≥2 EEG epochs per condition). Participant characteristics in the final sample are presented in Table 1. There were no significant differences between the clinical and control groups of the final sample in terms of chronological age (*F*(2,31) = 1.829, *p* > 0.10) nor between the ASD and ID groups in terms of developmental age (*t*(20) = − 0.033, *p* > 0.10).

**Table 1:** Participant characteristics in the final sample

	Group		
	ASD	ID	TD
N (boys:girls)	12 (11:1)	10 (9:1)	12 (9:3)
CA (years:months)	4:4 (0:11), 2:10–5:10	5:1 (1:1), 3:11–7:2	4:7 (0:10), 2:9–5:10
Developmental age (years: months)	2:7 (0:11), 1:2–4:2	2:7 (0:6), 1:10–3:4	
SCQ (cut-off 15)		9.11 (5.11), 4–18	3.25 (2.22), 0–7
ADOS-2			
SA	15.6 (3.1), 9–20		
RRB	5.3 (1.4), 3–8		
Comparison score	8.1 (1.5), 6–10		
ADI-R			
Social domain (cut-off 10)	19.8 (6.3), 6–28		
Communication domain (cut-off 8)	16 (2.3), 14–20^a^		
	10.5 (2.7), 7–14^b^		
Stereotypy domain (cut-off 3)	7.3 (2.7), 4–12		

All values are given as mean (SD), range

*ASD* autism spectrum disorder, *ID* intellectual disability, *TD* typical development, *CA* chronological age, *SCQ* Social Communication Questionnaire, *ADOS*-*2* Autism Diagnostic Observation Schedule 2, *SA* social affect, *RRB* restricted repetitive behaviour, *ADI*-*R* Autism Diagnostic Interview-Revised

^a^Verbal children (N = 4)

^b^Non-verbal children (N = 8)

Clinical ASD diagnoses were confirmed using the Autism Diagnostic Interview - Revised (ADI-R; Rutter et al. 2005) and the Autism Observation Schedule 2 (ADOS-2; Lord et al. 2012). According to the ADOS-2 comparison scores, the level of autism spectrum-related symptoms was high, and the ADI-R summary confirmed severe autistic behaviour (see Table 1). To ensure that children in the control groups did not have autistic features, their parents completed the Social Communication Questionnaire (SCQ; Rutter et al. 2003). Two children with ID exceeded the cut-off score of 15 points in the questionnaire, but the additional points were obtained from the scale for difficulties in communication rather than from the scale for difficulties in social interaction. Furthermore, these two children did not show autistic behaviour in clinical observations.

### Stimuli

**Fig. 1:**
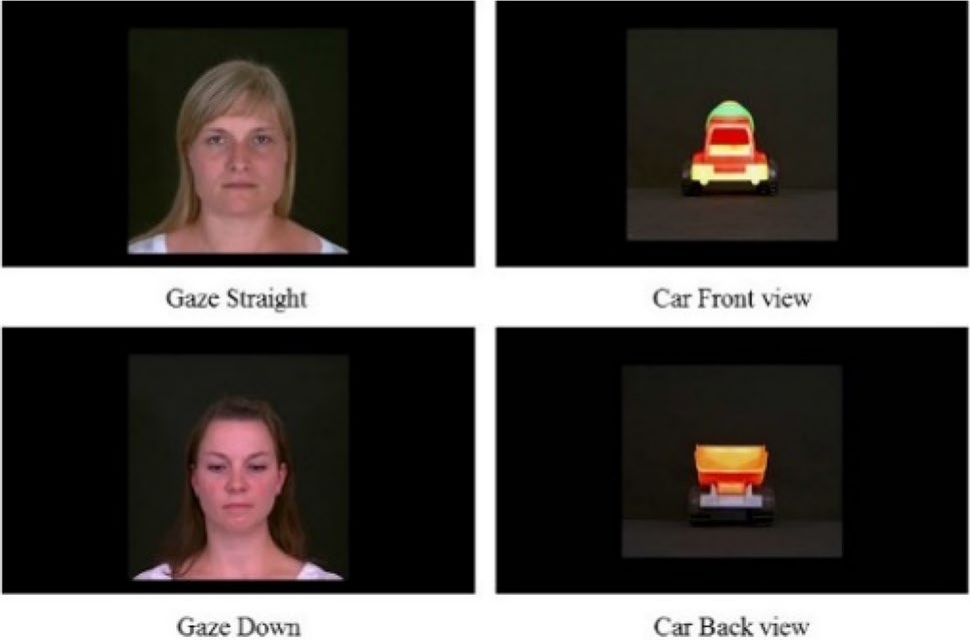
Still-picture examples of the video stimuli

Frontal views of faces with a neutral expression were videoed. Three women were instructed to pose with two different gaze directions: either while gazing straight ahead (direct gaze) or while gazing downwards (downcast gaze). The control stimuli comprised videos of three different toy cars with either a front or back view (see Fig. 1, for examples of still-picture video stimuli). Toy cars were chosen as control stimuli since they are familiar stimuli for the children and they have symmetrical appearance like faces. In addition, to match with the motion of the face stimuli (approach), movement is natural also for cars, unlike for many other control objects often used in face perception studies (e.g., houses).

Trials started with an attention grabber (a round-shaped moving figure with a sound effect) that was located on the screen in the same location as the eye region of the following face image. The trials were composed such that videos of faces and cars were static for the first 2 s, after which the faces started to loom towards children, thus creating the impression of an approaching person. In the car video stimuli, either the front end or the rear end of the car began to approach the child (depending on whether the video was filmed with a front or a back view of the car). The moving segment lasted for 3 s.

The vertical angle for the faces was 10° at the beginning of the trial and 17° at the end, while the vertical angle for the car stimuli measured 7° at the beginning and 13° at the end. The trials ended with a picture of a button signalling to the child that she/he could press a green or red button. This part of the task was aimed at engaging the child. Finally, a 1-s long reward animation appeared on the screen.

### Procedure

The experiment took place in a dimly lit room. First, the experimental procedure was carefully explained to the child with the help of picture cards, as per the practical guidelines set forth by Kylliäinen et al. (2014). Children sat within a viewing distance of approximately 60 cm from the screen. The task was a part of an experimental session that was approximately 1 h in length: about half of the children saw the task as the second task, while the remaining saw the task as their last one. It took the children approximately 10 min to complete the task. An experimenter sat/stood behind the child, whilst other experimenters and parents were at the other side of the room behind a wall. The experimental session was videotaped to enable the researcher to control stimulus presentation and to provide a record of the children’s movement and behaviour during the task.

There were 24 trials, including 6 trials of each stimulus type (direct gaze, downcast gaze, car with a front view, or car with a back view). Trials were controlled and presented using E-prime software (Psychology Software Distribution, Pittsburgh, Pennsylvania) in a random order with the constraint that the same stimulus type was presented no more than three times in a row. The inter-stimulus interval varied between 4–6 s. During the experiment, the experimenter monitored the child via a video feed and did not initiate the next trial unless the child was paying attention to the attention grabber on the screen. Short breaks between trials were allowed to help the children focus on the task.

### EEG Data Acquisition

Continuous EEG was recorded at 250 Hz using a high-density EGI Hydrocel 128-electrode net, Netamps 400 amplifier, and Netstation 4.5.1 software (Electrical Geodesics, Inc.). In the EGI recording system, impedances at or below 100 kΩ are considered acceptable based on the high input impedance of the EGI amplifiers (e.g., Richards, 2003). Sensor nets of different sizes were employed, and the one that most closely corresponded to the child’s head circumference was used.

Even though EEG was recorded from the whole surface, the analyses focused on specific channels in frontal areas. A subset of three individual electrodes on each hemisphere was chosen for each child depending on the exact placement of the net. The electrodes were chosen according to the 10–20 system of electrode placement for F3 and F4 (e.g., see Yang et al. 2007). The most common subset of three electrodes for the right hemisphere was 2, 3, 9 (N = 12), and the most common sets for the left hemisphere were 23, 26, 27 and 24, 27, 28 (both N = 10).

### Data Analysis

Stimulus-locked EEG data for trials were segmented offline into two 3000-ms segments to perform rudimentary corrections for blinks and for artefacts. The first segment covered 1000-ms pre-stimulus to 2000-ms post-stimulus (when the movement began) and the second segment covered 1000-ms to 4000-ms post-stimulus (when the picture had been moving for 2000 ms). EEG channels whose amplitudes exceeded 500-microvolts were excluded from further analyses and interpolated using the EEGLAB eeg interp function (Delorme and Makeig 2004). EEG in all channels were re-referenced to the average reference (i.e., the mean of all channels). Baseline-correction was applied by computing the mean of each segment and subtracting it from the EEG signal within each segment and eye movements and blinks were corrected using Independent Component Analysis (ICA, Delorme and Makeig 2004).

After ICA, the EEG data were segmented into two 1-s long epochs within each trial. The first epoch was 0–1000 ms from the beginning of each trial (the first second of the static phase of the trial), and the second epoch was 2000–3000 ms from the beginning of the trial (the first second of the moving phase of the trial). By splitting the trials into these two epochs, we were able to maximize the number of accepted epochs and were able to analyse the effect of stimulus motion more carefully. Together, there were 8 different conditions: static direct gaze, dynamic direct gaze, static downcast gaze, dynamic downcast gaze, static car with a front view, dynamic car with a front view, static car with a back view, and dynamic car with a back view.

At this point, epochs were rejected based on video- and EEG-based quality control using Eegtool (Kaatiala et al. 2013), an open access Matlab toolbox, and integrated video-based quality control with EEGLAB functions (Delorme and Makeig 2004) for EEG-signal analyses. Rejection criteria in the video-based analyses included overt emotional reactions; gross body, hand, or head movements; or any stereotypic movements. Epochs were also rejected if the child did not look at the screen. The remaining EEG artefacts were recognized with an amplitude criterion of 150 µV and were corrected by interpolation (spherical splines). However, if the number of bad EEG channels in an epoch exceeded 10% of the 128 electrode channels, the entire epoch was rejected.

The Fast Fourier Transform analysis (FFT)-transformed signal was used to calculate the mean of the alpha-activity power (µV^2^) in each electrode for every condition. Consistent with prior research in this age group, the frequency band of 6–10 Hz was used for defining alpha-activity (Marshall et al. 2002).

After calculating the means for each electrode for every condition, we calculated the mean power density values for the chosen subset of three electrodes on right and left hemisphere for each condition. To normalize the distributions, these means underwent a natural-log transform. For each condition, asymmetry scores were then calculated by subtracting the natural log-transformed mean value of the left-sided subset of electrodes from that of the right-sided subset of electrodes.

Owing to the inverse relationship between alpha-power and cortical activity (e.g., see Allen et al. 2004), positive values indicate a relatively greater alpha-power in the right frontal areas, suggesting relatively higher levels of cortical activity on the left side. Negative values indicate relatively higher levels of cortical activity on the right side.

The data from the participants were taken into the final analyses if there were at least two accepted epochs in each of the eight conditions. In total, in the ASD group, there was an average of 35 (range 23–48) accepted epochs per subject, of which 18 were from the static phases and 17 from the moving phases of the trials. In the ID group, the average number of accepted epochs was 32 (20–42), of which 16 were from the static phases and 16 from the moving phases. Among TD children, the average number of accepted epochs was 33 (23–42), of which 17 were from the static phases and 16 were from the moving phases. There were no statistically significant differences between the groups in the number of accepted epochs (*F*(2, 31) = 0.549, *p* > 0.05, *η*_*p*_^2^ = 0.034). Neither were there differences in the number of accepted trials for the static (*F*(2, 31) = 0.433, *p* > 0.05, *η*_*p*_^2^ = 0.027) or moving phases (*F*(2, 31) = 0.639, *p* > 0.05, *η*_*p*_^2^ = 0.040).

## Results

A four-way ANOVA with group (ASD, ID, TD) as a between-subject factor and stimulus (face, car), direction (direct gaze/frontal view, downcast gaze/back view) and movement (dynamic, static) as within-subject factors showed a trend for interaction between all four main effects (*F*(2, 31) = 3.264, *p* = 0.052, *η*_*p*_^2^ = 0.174). The ANOVA also showed a significant three-way interaction between group, direction, and movement (*F*(2, 31) = 3.526, *p* = 0.042, *η*_*p*_^2^ = 0.185), and a two-way interaction between group and movement (*F*(2, 31) = 3.813, *p* = 0.033, *η*_*p*_^2^ = 0.197). Other interactions or main effects were not significant.

**Fig. 2:**
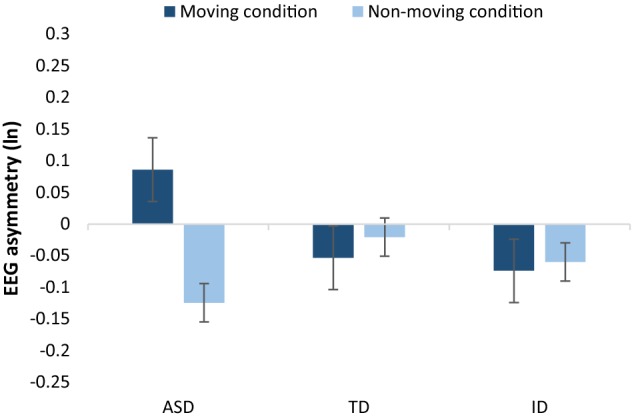
Mean (and SEM) frontal asymmetry scores as a function of stimulus movement and group (ln-transformed right-sided value—ln-transformed left-sided value). Positive values indicate relative left-sided, approach-related EEG activity and negative values indicate relative right-sided, avoidance-related EEG activity

Because of the interaction between group and movement, the effect of movement was first analysed separately for each group. For the ASD group, asymmetry scores were significantly higher for moving than for static stimuli (*t*(11) = 2.262, *p* = 0.045). For the other two groups, movement did not have a significant effect on asymmetry scores (TD children: *t*(11) = − 0.898, *p* > 0.05; ID children: *t*(9) = − 0.204, *p* > 0.05, see Fig. 2).

**Fig. 3:**
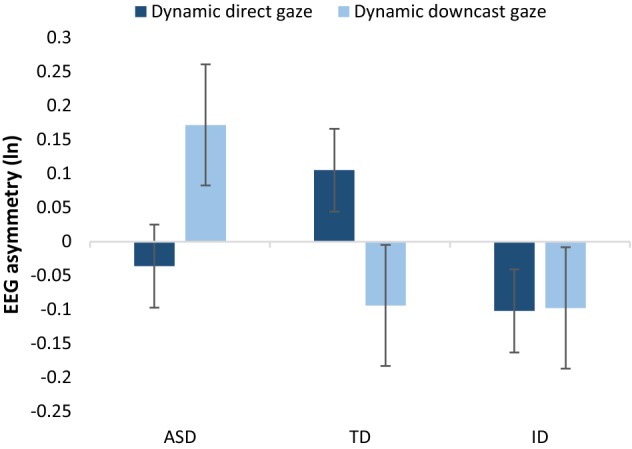
Mean (and SEM) frontal asymmetry scores as a function of dynamic gaze conditions and group (ln-transformed right-sided value – ln-transformed left-sided value). Positive values indicate relative left-sided, approach-related EEG activity and negative values indicate relative right-sided, avoidance-related EEG activity

In the subsequent analyses, the data related to periods when the stimuli were moving and static were separated. For the static stimuli, a three-way ANOVA (group x stimulus x direction) showed no significant main effects or interactions (*p* > 0.05 for all). However, for the dynamic stimuli, the same analysis showed a statistically significant interaction between group, stimulus, and direction (*F*(2, 31) = 6.099, *p* = 0.006, *η*_*p*_^2^ = 0.282), and between group and direction (*F*(2, 31) = 3.959, *p* = 0.029, *η*_*p*_^2^ = 0.203). Because of these interactions, we analysed the data for dynamic toy cars and dynamic gaze stimuli separately. For dynamic toy cars, a group x direction ANOVA showed no significant main effects or interaction (*p* > 0.05). In contrast, for dynamic gaze stimuli, the interaction between group and direction was significant (*F*(2, 31) = 7.961, *p* = 0.002, $\eta_{p}^{2}$ = 0.339). The main effects of group and direction were not significant (*p* > 0.05). Pairwise comparisons showed that, for typically developing children, the asymmetry scores were more positive for dynamic direct gaze than for dynamic downcast gaze (*t*(11) = 2.890, *p* = 0.015). In striking contrast to the result for TD children, in the ASD group, the asymmetry scores were more positive for downcast gaze than for direct gaze (*t*(11) =  − 5.006, *p* < 0.0001). In other words, seeing a face with downcast gaze elicited relatively greater left-sided, approach-related frontal activity compared to seeing a face with direct gaze (see Fig. 3). Children with intellectual disability did not show differences in asymmetry scores between dynamic direct and dynamic downcast gaze (*t*(9) = − 0.040, *p *> 0.05).

## Discussion

In the present study, we investigated approach- and avoidance-motivation-related frontal EEG asymmetry in response to direct and downcast gaze in young children with ASD and intellectual disability (ID) and in both chronologically and developmentally age-matched control children. The results of the present study revealed that in TD children, direct gaze elicited greater approach-related frontal activity than did downcast gaze. The pattern of activity was completely opposite in the children with ASD who showed greater approach-related activity in response to downcast gaze than to direct gaze. The control children with intellectual disability without ASD did not show differences in their frontal EEG activity patterns in response to direct versus downcast gaze.

The results showing greater approach-related frontal brain activity in response to direct gaze in 3- to 6-year-old TD children are in line with the results of our previous studies showing similar patterns in school-aged children (Kylliäinen et al. 2012) and healthy adults (Hietanen et al. 2008; Pönkänen et al. 2011). The consistency of this response pattern across age groups supports the view that differential sensitivity to eye contact is a salient and potentially stable characteristic of typical development. Previous studies have shown selective enhancement of frontal gamma-band activity to direct gaze in infants, suggesting an early-emerging neural specialization for the processing of this cue (Grossmann et al. 2007; Parise and Csibra 2013). There are also indications that infants and young children may interpret direct gaze as an ostensive signal that identifies them as a recipient of active social communication (e.g., gaze cueing or pointing gestures, Parise and Csibra 2013; Senju and Csibra 2008). Our findings are consistent with these views and add to them by showing that the response to eye contact in children may also engage affective-motivational brain systems and a pattern of EEG activity that has been repeatedly linked with approach-related behavioural tendencies.

The absence of an enhanced approach-related frontal activity in response to direct gaze in the currently studied young children with ASD and ID is consistent with the findings of our previous study involving school-aged, cognitively able children with ASD (Kylliäinen et al. 2012). The current findings are also in line with the findings of our recent study (Helminen et al. 2017), which involved mostly the same sample of children as in the present study and showed that young children with severe ASD do not show an enhanced heart rate orienting response to direct gaze, whereas TD children and children with ID without ASD do. We interpreted (Helminen et al. 2017) these findings as being consistent with the hypothesis that ASD is associated with an absence of enhanced motivational and attentional responses to eye contact and, as a consequence, children with ASD may passively omit attending to these cues (cf. Senju et al. 2005). Thus, abnormalities in attention to eyes in ASD may arise from the absence of typical responses to eye contact instead of an active avoidance of this social cue. Recently, Moriuchi et al. (2017) also showed that young children with ASD do not look away from the eyes any faster than do TD children, which is consistent with this view.

However, the results of the present study suggest that instead of only a lack of age-typical response, young children with ASD and ID may be characterized by a more complex pattern of abnormalities in response to eye contact. In contrast to the results from school-aged children with ASD who did not have any difference in frontal EEG asymmetry responses between direct gaze and closed eyes (Kylliäinen et al. 2012), here we found that gaze direction indeed has an effect on frontal EEG asymmetry scores in these young children with ASD: the downcast gaze elicited relatively greater left-sided, approach-related frontal activity than did the direct gaze. So, how to interpret this result? Why would downcast gaze be more approachable than direct gaze for children with ASD? We would like to point out that, unlike the other groups of children, the children with ASD showed, in general, greater approach-related frontal EEG activity in response to the dynamic conditions than to the static conditions (cf., Fig. 2). Thus, stimulus motion as such had an impact on the ASD children’s frontal EEG asymmetry. Now, one way to interpret the findings in ASD children is to argue that the typical pattern of approach-related frontal activity in response to dynamic stimuli was decreased when the stimulus was a face with direct gaze. Thus, in our view, we cannot entirely rule out the possibility that direct gaze evokes avoidance-related responses in young children with ASD and ID.

In our statistical analyses, we confined ourselves to within-group comparison. When investigating task-dependent changes in frontal EEG asymmetry in different groups of participants, we feel that it is safest to compare the patterns of responses to different stimulus conditions *within* each group instead of comparing responses to a given condition (e.g., direct gaze) between the groups. For example, the resting state frontal EEG asymmetry shows great individual and situational variation in individuals with ASD (Burnette et al. 2011; Heunis et al. 2016; Sutton et al. 2005; Wang et al. 2013). Thus, the direct, stimulus-related between-group comparisons would be complicated due to the possible differences in the baseline activity patterns. Without a similar baseline, it is impossible to interpret if even an identical EEG response (asymmetry score) to a given stimulus indicates similar or different approach-avoidance related brain response to this stimulus between participants from two different groups.

Rather surprisingly, the children who had ID without ASD did not show a difference in their responses to direct and downcast gaze stimuli. Both gaze directions elicited relatively greater right-sided, avoidance-related frontal asymmetry which was against our hypothesis of relatively greater left-sided frontal activity in response to direct gaze. Previous literature has suggested that a stimulus moving towards an observer is an indicator of threat (Shiff et al. 1962). Stimuli looming towards the perceiver have been shown to trigger avoidance-related defensive responses (i.e., pulling the head away from the stimulus, placing hands over the mouth) already in 2- to 11-week-old infants (Ball and Tronick 1971). It is possible that, in the ID group, the stimulus movement towards an observer itself captured their attention and elicited the avoidance-related frontal EEG asymmetry. It might be that the approaching and self-relevant movement was a reason for avoidance-related frontal EEG asymmetry in the downcast gaze condition in TD children as well. It is further possible that cognitive deficits in children with ID (e.g., spared global but impaired local visual processing, Bihrle et al. 1989) may lead to a situation in which salient stimulus motion overshadows the processing of facial features (gaze direction) and explains the lack of differential motivation-related brain responses to these cues. Further research would be needed, however, to clarify whether approaching movement as such elicits avoidance motivation, independent of factors like gaze direction in children with lower developmental levels.

A limitation that should be noted, when making conclusions of the effect of movement in the present experiment, is that the static and the dynamic phases were parts of the same trial and appeared always in the same order, i.e., the former preceded the latter. The stimulus was first static for two seconds and only after that started to move towards the observer. Consequently, we were able to maintain the children’s attention on the screen and made the stimuli look more dynamic and natural than static images. In children with ID and TD children, there were no differences in the brain activity between static and dynamic conditions, when data from all the stimuli were pooled. The children with ASD were the only ones who showed differences in their responses to static and dynamic stimuli. It is possible that the children without ASD learnt to anticipate that the stimulus would loom forward right after the static phase. The anticipation could have affected the neural activity in the static phase of the trial and therefore the difference between the static and dynamic conditions was reduced. In contrast, the children with ASD may have lacked this effect of movement anticipation. Previous studies have shown that individuals with ASD have deficits in anticipating their own movements according to the movements of their social counterparts (Brisson et al. 2012; Schmitz et al. 2003) and in anticipating social sequences (Palumbo et al. 2015; Zalla et al. 2010). In future studies, it is important to present the dynamic and static conditions in different trials to better verify the effect of stimulus movement on alpha-asymmetry.

Furthermore, the previous studies have used only pictorial stimuli, most likely because of difficulties in getting individuals with severe ASD to look at faces and guiding them through demanding laboratory settings. In the present study, as in the studies by Moriuchi et al. (2017) and Helminen et al. (2017), the children were cued to look at the eyes by task properties. This has, however, not been the case in all studies of abnormal gaze processing in ASD (Kaartinen et al. 2012; Stagg et al. 2013), and it is therefore difficult to conclude whether all results are related to the possible gaze aversion rather than face aversion. Recently, Hadjikhani et al. (2017) demonstrated that when constrained to look in the eyes, individuals with ASD show abnormally high activation in the subcortical social brain network (including amygdala); the effect was not as strong when participants were freely viewing the face. Therefore, in future studies, it should be specified whether the aim is to investigate aversion to faces in general, or aversion to eyes and eye contact in particular. In future studies, it would also be essential to investigate how even more naturalistic stimuli than in the current study, such as a live person, affect the frontal EEG activity in children with ASD.

To conclude, this study provides evidence for early abnormalities in motivational responses to eye contact in autistic development. The greater approach-motivation related frontal activity to direct gaze, which appears to be a consistent characteristic of typical development, was not found in children with ASD or ID. In contrast, children with ASD exhibited more approach-related EEG activity to downcast gaze than to direct gaze, whereas children with ID without ASD did not show any difference in their responses to direct and downcast gaze. While some of the results in ASD and ID were unexpected and should be interpreted with caution, they point to group-specific abnormalities in response to social cues in children with ASD and ID, and call for further research on the mechanisms that may explain these differences and the associated difficulties in social behaviour.

## References

[CR1] Allen J, Coan J, Nazarian M (2004). Issues and assumptions on the road from raw signals to metrics of frontal EEG asymmetry in emotion. Biological Psychology.

[CR2] Ball W, Tronick E (1971). Infant responses to impending collision: Optical and real. Science.

[CR3] Batki A, Baron-Cohen S, Wheelwright S, Connellan J, Ahluwalia J (2000). Is there an innate gaze module? Evidence from human neonates. Infant Behavior and Development.

[CR4] Bayley N (2006). Bayley scales of infant and toddler development.

[CR5] Bihrle AM, Bellugi U, Delis D, Marks S (1989). Seeing either the forest or the trees: Dissociation in visuospatial processing. Brain and Cognition.

[CR6] Brisson J, Warreyn P, Serres J, Foussier S, Adrien-Louis J (2012). Motor anticipation failure in infants with autism: a retrospective analysis of feeding situations. Autism.

[CR7] Burnette CP, Henderson HA, Inge AP, Zahka NE, Schwartz CB, Mundy PC (2011). Anterior EEG asymmetry and the modifier model of autism. Journal of Autism and Developmental Disorders.

[CR8] Davidson R, Izard CE, Kagan J, Zajonc RB (1984). Affect, cognition and hemispheric lateralization. Emotion, cognition, and behaviour.

[CR9] Dawson G, Webb SJ, McPartland J (2005). Understanding the nature of face processing impairment in autism: Insights from behavioral and electrophysiological studies. Developmental Neurophysiology.

[CR10] Delorme A, Makeig S (2004). EEGLAB: an open source toolbox for analysis of single-trial EEG dynamics including independent component analysis. Journal of Neuroscience Methods.

[CR11] Elsabbagh M, Mercure E, Hudry K, Chandler S, Pasco G, Charman T (2012). Infant neural sensitivity to eye gaze predicts characteristics of autism at two years. Current Biology.

[CR12] Farroni T, Csibra G, Simon F, Johnson M (2002). Eye contact detection in humans from birth. Proceedings of the National academy of Sciences of the United States of America.

[CR13] Farroni T, Menon E, Johnson M (2006). Factors influencing newborns’ preference for faces with eye contact. Journal of Experimental Child Psychology.

[CR14] Grossmann T, Johnson MH, Farroni T, Csibra G (2007). Social perception in the infant brain: gamma oscillatory activity in response to eye gaze. Social Cognitive and Affective Neuroscience.

[CR15] Hadjikhani N, Johnels JÅ, Zürcher NR, Lassalle A, Guillon Q, Hippolyte L (2017). Look me in the eyes: constraining gaze in the eye-region provokes abnormally high subcortical activation in autism. Scientific Reports.

[CR16] Harmon-Jones E, Gable PA (2018). On the role of asymmetric frontal cortical activity in approach and withdrawal motivation: An updated review of the evidence. Psychophysiology.

[CR17] Helminen TM, Leppänen JM, Eriksson K, Luoma A, Hietanen JK, Kylliäinen A (2017). Atypical physiological orienting to direct gaze in low-functioning children with autism spectrum disorder. Autism Research.

[CR18] Heunis TM, Aldrich C, de Vries PJ (2016). Recent advances in resting-state electroencephalography biomarkers for autism spectrum disorder: A review of methodological and clinical challenges. Pediatric Neurology.

[CR19] Hietanen JK, Leppänen JM, Peltola MJ, Linna-aho K, Ruuhiala HJ (2008). Seeing direct and averted gaze activates the approach-avoidance motivational brain systems. Neuropsychologia.

[CR20] Itier RJ, Batty M (2009). Neural bases of eye and gaze processing: the core of social cognition. Neuroscience and Biobehavioral Reviews.

[CR21] Jack A, Pelphrey K (2017). Annual research review: understudied populations within the autism spectrum - current trends and future directions in neuroimaging research. Journal of Child Psychology and Psychiatry.

[CR22] Jones W, Klin A (2013). Attention to eyes is present but in decline in 2–6-month-old infants later diagnosed with autism. Nature.

[CR23] Joseph R, Ehrman K, McNally R, Keehn B (2008). Affective response to eye contact and face recognition ability in children with ASD. Journal of International Neuropsychological Society.

[CR24] Kaartinen M, Puura K, Mäkelä T, Rannisto M, Lemponen R, Helminen M, Salmelin R, Himanen S-L, Hietanen JK (2012). Autonomic arousal to direct gaze correlates with social impairments among children with ASD. Journal of Autism and Developmental Disorders.

[CR25] Kaatiala J, Yrttiaho S, Forssman L, Perdue K, Leppänen JM (2013). A graphical user interface for infant ERP analysis. Behavior Research Methods.

[CR26] Kylliäinen A, Hietanen JK (2006). Skin conductance responses to another person’s gaze in children with autism. Journal of Autism and Developmental Disorders.

[CR27] Kylliäinen A, Jones EJH, Gomot M, Warreyn P, Falck-Ytter T (2014). Practical guidelines for studying young children with autism spectrum disorder in psychophysiological experiments. Review Journal of Autism and Developmental Disorders.

[CR28] Kylliäinen A, Wallace S, Coutanche M, Leppänen JM, Cusack J, Bailey AJ, Hietanen JK (2012). Affective-motivational brain responses to direct gaze in children with autism spectrum disorder. Journal of Child Psychology and Psychiatry.

[CR29] Lord C, Rutter M, DiLavore P, Risi S, Gotham K, Bishop S (2012). Autism diagnostic observation schedule, second edition (ADOS-2).

[CR30] Maestro S, Muratori F, Cavallaro M, Pecini C, Cesari A, Paziente A, Stern D, Golse B, Palacio-Espasa F (2005). How young children treat objects and people: An empirical study of the first year of life in autism. Child Psychiatry and Human Development.

[CR31] Marshall P, Bar-Haim Y, Fox N (2002). Development of the EEG from 5 months to 4 years of age. Clinical Neurophysiology.

[CR32] Moriuchi JM, Klin A, Jones W (2017). Mechanisms of diminished attention to eyes in autism. American Journal of Psychiatry.

[CR33] Nuske H, Vivanti G, Dissanayake C (2015). No evidence of emotional dysregulation or aversion to mutual gaze in preschoolers with autism spectrum disorder: An eye-tracking pupillometry study. Journal of Autism and Developmental Disorders.

[CR34] Osterling J, Dawson G (1994). Early recognition of children with autism: a study of first birthday home videotapes. Journal of Autism and Developmental Disorders.

[CR35] Osterling JA, Dawson G, Munson JA (2002). Early recognition of 1-year old infants with autism spectrum disorder versus mental retardation. Development and Psychopathology.

[CR36] Palumbo L, Burnett HG, Jellema T (2015). Atypical emotional anticipation in high-functioning autism. Molecular Autism.

[CR37] Parise E, Csibra G (2013). Neural responses to multimodal ostensive signals in 5-month-old infants. PLoS ONE.

[CR38] Pönkänen L, Peltola M, Hietanen J (2011). The observer observed: frontal EEG asymmetry and autonomic responses differentiate between another person’s direct and averted gaze when the face is seen live. International Journal of Psychophysiology.

[CR39] Richards JE (2003). Cortical sources of event-related potentials in the prosaccade and antisaccade task. Psychophysiology.

[CR40] Rutter M, Bailey A, Lord C (2003). SCQ: Social Communication Questionnaire.

[CR41] Rutter M, Le Couteur A, Lord C, Faggioli R (2005). ADI-R: Autism diagnostic interview-revised: Manual.

[CR42] Saitovitch A, Bargiacchi A, Chabane N, Phillipe A, Brunelle F, Boddaert N (2013). Studying gaze abnormalities in autism: Which type of stimulus to use?. Open Journal of Psychiatry.

[CR43] Schmitz C, Martineau J, Barthélémy C, Assaiante C (2003). Motor control and children with autism: deficit of anticipatory function?. Neuroscience Letters.

[CR44] Senju A (2013). Atypical development of spontaneous social cognition in autism spectrum disorders. Brain and Development.

[CR45] Senju A, Csibra G (2008). Gaze following in human infants depends on communicative signals. Current Biology.

[CR46] Senju A, Hasegawa T, Tojo Y (2005). Does perceived direct gaze boost detection in adults and children with and without autism?. The stare-in-the-crowd effect revisited. Visual Cognition.

[CR47] Senju A, Johnson MH (2009). Atypical eye contact in autism: Models, mechanisms and development. Neuroscience and Biobehavioral Reviews.

[CR48] Shiff W, Caviness J, Gibson J (1962). Persistent fear responses in rhesus monkeys to the optical stimulus of “looming”. Science.

[CR49] Stagg S, Davis R, Heaton P (2013). Associations between language development and skin conductance responses to faces and eye gaze in children with autism spectrum disorder. Journal of Autism and Developmental Disorders.

[CR50] Sutton SK, Burnette CP, Mundy PC, Meyer J, Vaughan A, Sanders C, Yale M (2005). Resting cortical brain activity and social behavior in higher functioning children with autism. Journal of Child Psychology and Psychiatry.

[CR51] Uusberg H, Allik J, Hietanen JK (2015). Eye contact reveals a relationship between neuroticism and anterior EEG asymmetry. Neuropsychologia.

[CR52] Wang J, Barstein J, Ethridge LE, Mosconi MW, Takarae Y, Sweeney JA (2013). Resting state EEG abnormalities in autism spectrum disorders. Journal of Neurodevelopmental Disorders.

[CR53] Wechsler D (2002). The Wechsler Primary and Preschool Scale for Intelligence (3rd revision).

[CR54] Werner E, Dawson G, Munson J, Osterling J (2005). Variation in early developmental course in autism and its relation with behavioral outcome at 3–4 years of age. Journal of Autism and Developmental Disorders.

[CR55] Yang C, Perfetti C, Schmalhofer F (2007). Event-related potential indicators of text integration across sentence boundaries. Journal of Experimental Psychology: Learning, Memory, and Cognition.

[CR56] Zalla T, Labruyère N, Clément A, Georgieff N (2010). Predicting ensuing actions in children and adolescents with autism spectrum disorders. Experimental Brain Research.

